# Magnetically Assisted Bilayer Composites for Soft Bending Actuators

**DOI:** 10.3390/ma10060646

**Published:** 2017-06-12

**Authors:** Sung-Hwan Jang, Seon-Hong Na, Yong-Lae Park

**Affiliations:** 1 Robotics Institute, School of Computer Science, Carnegie Mellon University, Pittsburgh, PA 14213, USA; sj2527@columbia.edu; 2 Novel Aerospace Materials, Faculty of Aerospace Engineering, Delft University of Technology, Kluyverweg 1, Delft 2629 HS, The Netherlands; 3 Department of Civil Engineering and Engineering Mechanics, Columbia University, New York, NY 10027, USA; s.na@columbia.edu; 4 Department of Mechanical and Aerospace Engineering, Seoul National University, Seoul 08826, Korea; 5 Institute of Advanced Machines and Design, Seoul National University, Seoul 08826, Korea

**Keywords:** soft actuator, pneumatic bending actuator, bilayer composite, non-uniform magnetic field, ferromagnetic particles

## Abstract

This article presents a soft pneumatic bending actuator using a magnetically assisted bilayer composite composed of silicone polymer and ferromagnetic particles. Bilayer composites were fabricated by mixing ferromagnetic particles to a prepolymer state of silicone in a mold and asymmetrically distributed them by applying a strong non-uniform magnetic field to one side of the mold during the curing process. The biased magnetic field induces sedimentation of the ferromagnetic particles toward one side of the structure. The nonhomogeneous distribution of the particles induces bending of the structure when inflated, as a result of asymmetric stiffness of the composite. The bilayer composites were then characterized with a scanning electron microscopy and thermogravimetric analysis. The bending performance and the axial expansion of the actuator were discussed for manipulation applications in soft robotics and bioengineering. The magnetically assisted manufacturing process for the soft bending actuator is a promising technique for various applications in soft robotics.

## 1. Introduction

Soft actuators have been widely used in many areas, such as robotics and biomedical engineering, due to their simple structures with flexibility and relatively high power density [1,2,3,4]. Bending motions in soft actuators generally can be achieved by changing the geometry and/or material properties by adding heterogeneous materials with different stiffness to the base polymer structure [5,6,7,8,9]. Niiyama et al. have developed pouch motors that could contract or bend by thermally bonding non-stretchable polymer films [10,11]. Gong et al. have used individually inflatable multiple air chambers embedded in a soft robotic arm for multi-directional bending [12]. Chang et al. have proposed a fluidic bending actuator by bonding two materials with different mechanical properties [13]. Gorissen et al. have developed a bending microactuator based on an asymmetric geometry of a thin film structure [14]. Udupa et al. have tried to maximize the bending performance by introducing an asymmetric structure with a bellow [15]. Paek et al. have developed soft bending tentacles made of elastomeric microtubes that have non-uniform wall thicknesses [16]. However, these approaches require tedious manual steps in fabrication and also complex designs. Moreover, it sometimes induces high stress concentrations at the interface between two different materials when bonded together [14].

A magnetically assisted fabrication is a promising method for enhancing mechanical properties and electrical and thermal conductivities of the base material by introducing ferromagnetic particles in the base structure [17,18,19,20,21]. An external magnetic field makes the particles move to specific directions through either attractive or repulsive interactions between the particles. When a non-uniform magnetic field is applied, it attracts ferromagnetic particles based on the gradient of the magnetic field. Especially, in a prepolymer state, magnetic particles can easily move with a relatively weak magnetic field because of the low viscosity of the polymer [22,23]. Thus, varied gradients of a magnetic field can achieve a stiffness gradient in a composite, which can be the main mechanism for a bending actuator. Also, the magnetically assisted technique enables simple fabrication of bilayer composites, alleviating stress concentrations between thermally or chemically bonded layers, frequently introduced in traditional fabrication methods. 

In this study, we propose a pneumatic soft bending actuator composed of a bilayer composite fabricated using a non-uniform magnetic field. The bilayer composite concentrated with nickel particles was prepared and characterized using an optical microscope and a scanning electron microscope (SEM) and then thermogravimetrically analyzed. The bending performance of the bilayer composite was tested in terms of bending angle and axial expansion by applying varied air pressures into the chamber of the actuator.

## 2. Materials and Methods

### 2.1. Actuator Materials

A highly stretchable silicone elastomer (Dragon Skin 10, Smooth-On, Easton, PA, USA) was obtained. Its density is approximately 1.05 g/cm^3^ and the curing time is about 24 h at room temperature. Nickel particles and ferromagnetic particles, were purchased from NOVAMET (Wyckoff, NJ, USA). Their average diameters and density were 12.0 μm and 8.5 g/cm^3^, respectively. Note that the density of nickel particle is approximately eight times larger than that of the silicone polymer.

### 2.2. Fabrication

**Figure 1:**
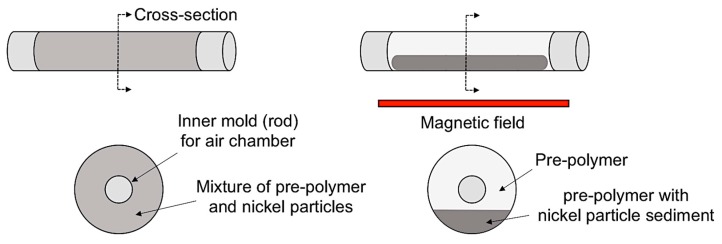
Schematic for fabrication of bilayer composite by using non-uniform magnetic field.

Figure 1 shows a schematic of fabrication for the bilayer bending actuator using a non-uniform magnetic field. First, Part A and Part B of Dragon Skin 10 were mixed with 1:1 by the weight ratio. Then, nickel particles were uniformly mixed with a centrifugal planetary mixer (ARE 301, Thinky Corporation, Tokyo, Japan) at 2000 rpm for 3 min. In this study, different concentrations of nickel particles (0, 0.5, 2.5, 5.0 wt %) were used to investigate their influences on the bending performance. After full dispersion, the mixture was degassed in a vacuum for 10 min to remove air bubbles. Then, the mixture was poured into a cylindrical mold and a gradient magnetic field generated by neodymium magnets (4760 gauss of surface field) was applied only at the bottom of the mold to attract nickel particles for about 10 min, as shown in Figure 1 (right). The mold was then cured in a convection oven at 60 °C for one hour. This magnetically assisted technique makes the fabrication simple, since it does not require any additional steps for preparation of multiple materials, bonding of cured layers, and change of geometries to control structural eccentricity.

**Figure 2:**
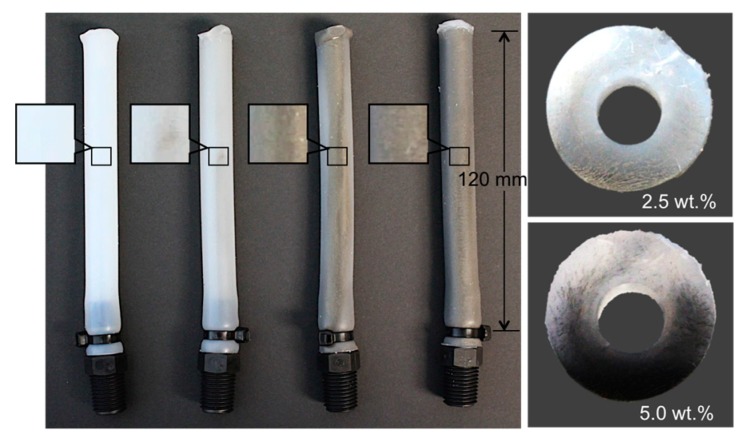
Bilayer composite soft bending actuators with different concentrations of nickel particles: 0, 0.5, 2.5, and 5.0 wt % (from left to right). The increased nickel particle concentrations are shown with color changes.

Figure 2 shows four different actuator samples made of bilayer composites with different particle concentrations. All the samples have outer diameters of 12.5 mm, inner diameters of 5 mm, and lengths of 120 mm. While one end of each tube was closed, the other end was connected to a tube fitting through which compressed air was injected. The overall weights of the bilayer composites increased with higher particle concentrations due to the density increase from the nickel particles.

### 2.3. Characterization

The morphology of the bilayer composite was investigated using a SEM (FEI Sirion 600, JEOL, Tokyo, Japan) to observe the distribution of the nickel particles in the polymer matrix. Thermogravimetric analysis was also conducted by using a high-resolution analyzer (TGA 2950, TA Instruments, New Castle, DE, USA) to measure the weight of the particle sediment in the composite. Samples of 20 mg of cured polymer from the composite were heated from 30 to 800 °C at a rate of 20 °C/min. Although it has been reported that nano-sized nickel particles oxide at high temperature and slightly increase the weight [24,25], the weight change from high temperature oxidation was negligible in our experiment, and the oxidation effect was not taken into account in calculating the particle concentrations. For actuation performance, the bilayer composites were inflated by injecting air into the chambers. The air pressure was increased until the actuator mechanically failed. During actuation, the bending angles and axial expansions were measured using an open source image-processing program (Image J 1.48, Bethesda, MD, USA).

## 3. Results and Discussion

### 3.1. Microstructure of Bilayer Composite

**Figure 3:**
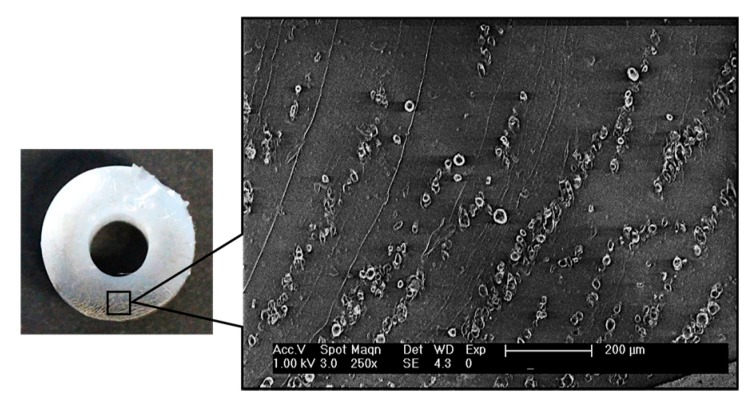
Cross-section of bilayer composite with 2.5 wt % nickel particles and SEM image of a particle-rich area showing aligned particle distribution.

The influence of the non-uniform magnetic field on the particle sedimentation of the bilayer composites was investigated by a SEM. Figure 3 shows the cross-section of a bilayer composite containing 2.5 wt % of nickel particles. It is clearly seen that the composite is divided into two different sections. This is because most of the nickel particles in a prepolymer state moved to the region with the stronger magnetic field due to the gradient in the field, that is that is $F_{mag}=\mu_{0}V(M\cdot \nabla )H$, where $\mu_{0}$ is the permeability in vacuum, V is the volume of the particle, and M is the magnetization in a given magnetic field H [26,27]. When particles are attracted by a magnetic field, they not only randomly aggregate but also align, making particle chains, as shown in the zoomed-in area in Figure 3. It is generally known that a composite with aligned particles show higher stiffness than one with randomly dispersed particles even though the particle concentrations of the two are the same [28,29]. Therefore, use of a magnetic field during fabrication has advantages of not only expediting and simplifying the process but also improving material properties in our application.

There are mixtures of aligned and aggregated nickel particles in the bottom area. This particle rich region shows a higher stiffness than the pure polymer region.

**Figure 4:**
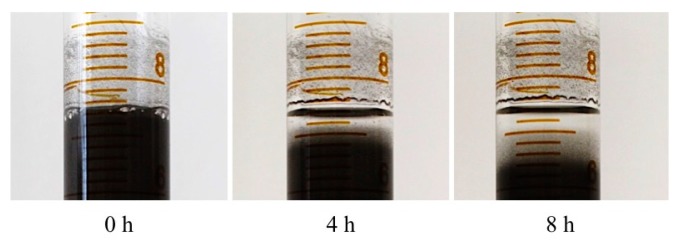
Particle sedimentation in PDMS that has similar viscosity but is clear only by gravity without a magnetic field.

To evaluate the contribution of the magnetic assistance to particle sedimentation, degrees of sedimentation were inspected in a prepolymer mixed with nickel particles (5.0 wt %) without applying a magnetic field. For a clear visual inspection, polydimethylsiloxane (PDMS) (Sylgard 184 Silicone Elastomer Kit, Dow Corning, Midland, MI, USA) was used instead of Dragon Skin 10 in this experiment, since PDMS was optically transparent while showing a similar viscosity in a prepolymer state. Although sedimentation occurred only by gravity due to the high density difference between the particles and the polymer, it required a highly prolonged period of time (about eight hours) compared to the time in the magnetically assisted technique (about 30 s), as shown in Figure 4. 

### 3.2. Thermogravimetric Analysis of Bilayer Composite

**Figure 5:**
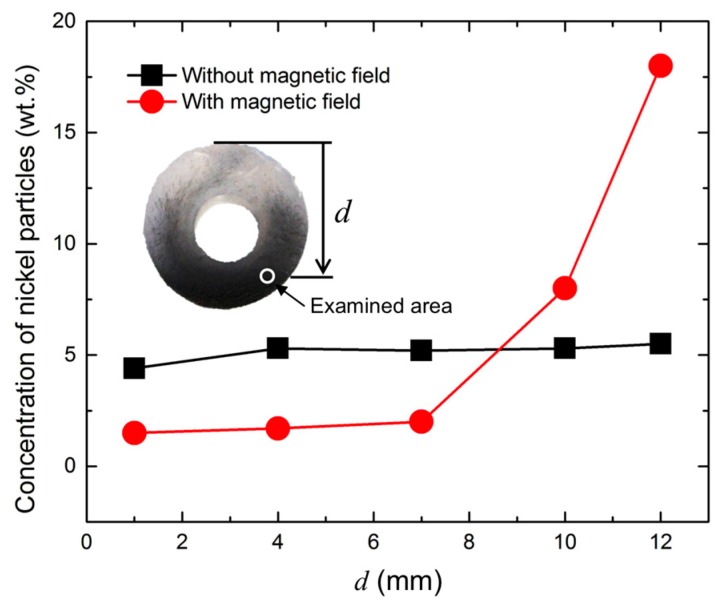
Result of thermogravimetric analysis of bilayer composite (5.0 wt % of nickel particles) with/without magnetic field.

The thermogravimetric analysis was performed for quantitatively analyzing the bilayer composites on the particle distributions in the polymer matrix. Since the silicone elastomer starts to degrade at 380 °C and the corresponding residue yield is 45% at 600 °C, the particle concentrations in the composites were calculated based on the TGA results of pure elastomer [30]. Figure 5 presents the result of the thermogravimetric analysis of a bilayer composite with 5.0 wt % nickel particles with/without a magnetic field. As expected, the composite without a magnetic field showed homogenous distribution of nickel particles throughout the matrix. However, in the composite with a non-uniform magnetic field, most of the nickel particles were deposited at the bottom of the structure, resulting in a clear bilayer structure. The concentration of the nickel particles increased as the vertical location of the examined area approached the bottom of the structure, as shown in Figure 5. The concentration of nickel particles in the particle-rich area is three times higher than that in a randomly dispersed composite. In addition, the composite with a higher concentration of nickel particles showed higher sediment thickness although a small amount of particle residues still remaining in the upper part due to the high viscosity of the polymer. 

### 3.3. Bending Performance of Soft Bilayer Actautor

**Figure 6:**
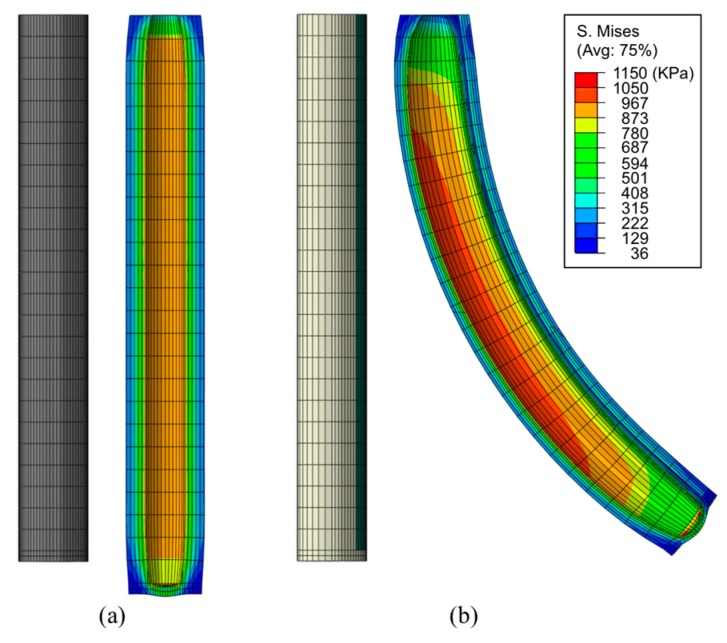
Effect of particle sedimentation on bending performance of soft bending actuator: (**a**) soft bending actuator with uniformly dispersed particles; (**b**) Soft bending actuator with particle sedimentation. (Color codes represent von Mises stress.)

**Figure 7:**
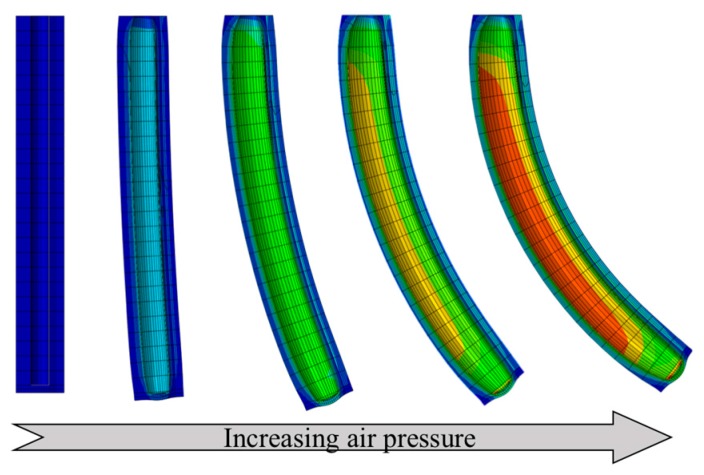
Bending performance of soft bilayer actuator under applied air pressure.

A bilayer composite bends from the combined effect of an end moment that develops at a free end due to the eccentricity and the differential expansion of the top and bottom layers [3,15,31]. The torque equilibrium can be obtained from $M_{a}=M_{\theta }$, where $M_{a}$ is the bending moment by the internal air pressure, and $M_{\theta }$ is the combined moment of the stresses on the top and the bottom layers. Before testing the actuators, three-dimensional finite element analysis (FEA) was conducted to simulate the bending behavior using a commercial FEA software package (ABAQUS/Standard, Simulia, Dassault Systemes, Providence, RI, USA). The material properties of a pure polymer tube and a polymer tube with embedded nickel particles were used for the characterizing both experimentally and in simulation. The pure polymer was simulated based on an incompressible hyperelastic model (neo-Hookean model) for its nonlinear behavior. For the particle embedded composite, we assumed that the composite was homogenous although the actual composite showed non-homogeneity of particle distribution for simplicity in simulation and simulated it based on a general rule of mixtures using an elastic model. One end of the actuator was fixed and air pressure in the chamber was increased. Figure 6 shows the behavior of the bilayer composite for bending performance. In the composite with homogenous particle distribution, shown in Figure 6a, only axial expansion was observed under the applied air pressure due to the uniform stiffness of the composite. However, both the bending and expansion were observed in the bilayer composite, as shown in Figure 6b. Figure 7 shows the bending performance of the bilayer composite under different air pressure levels, obtained from the FEM analysis. The actuator bent in the direction of the nickel particle composite layer due to the stiffness difference. 

**Figure 8:**
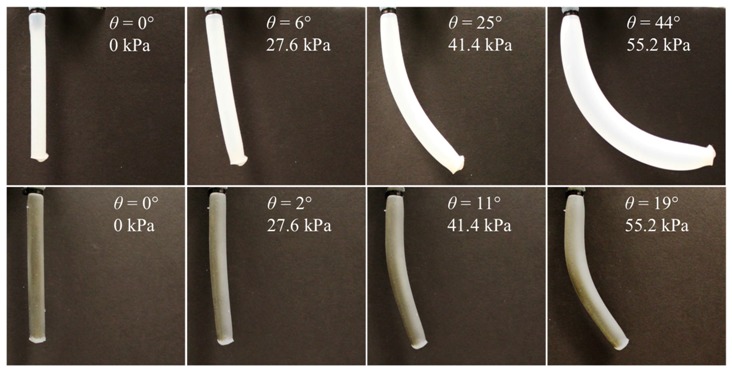
Bending performance test results of soft bilayer actuators with 0.5 wt % (**top**) and 5.0 wt % (**bottom**) nickel particle concentrations.

For experimentally analyzing the bending performance, the bending angles of the actuator were measured using the images taken during actuation, as shown in Figure 8. In this analysis, the bending angle *θ* was defined as the angle between the vertical line of the original shape of the actuator and the straight line that connected the base and the end point of the bent actuator. Figure 8 compares the bending behaviors of the actuators made of bilayer composites with two different nickel particle concentrations, 0.5 wt % and 5 wt %, with varied air pressure levels. It is noticed that the soft actuator with a lower particle concentration bends and axially expands more than that with a higher concentration. This is because the actuator with higher density requires additional force for both bending and expansion.

**Figure 9:**
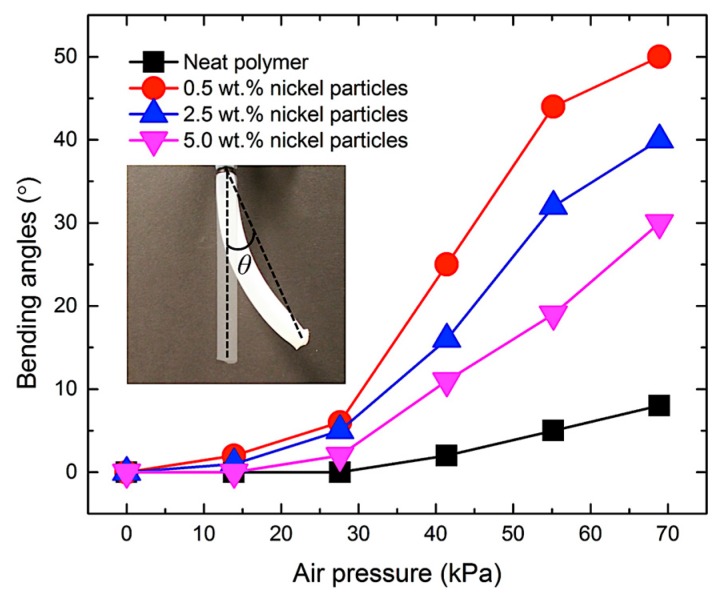
Bending angles of soft bending actuator as a function of air pressure.

**Figure 10:**
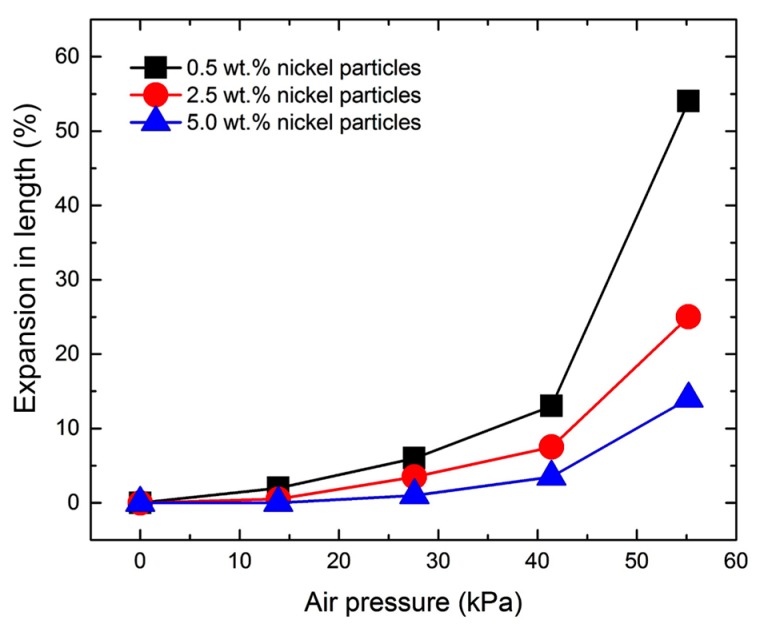
Normalized length expansion of soft bending actuator as a function of applied air pressure.

Figure 9 shows the representative bending angle changes with different particle concentrations for varied air pressure levels. The actuator with neat polymer showed lowest angle changes in bending, since the air chamber is located along the neutral axis and also the material is homogenous. Although small bending was observed in this actuator at a high air pressure, it is mainly from asymmetry of the structure caused by imperfection in manufacturing. All the composites with nickel particles showed relatively large bending angle changes. The actuation mechanism is to utilize an asymmetric structure based on the stiffness difference in a bilayer composite made by a non-uniform magnetic field during fabrication. It was also observed, in Figure 9, that the composites with low particle concentrations made larger bending angles than those with high concentrations. Although the asymmetric particle distribution in the composite enabled bending of the structure, increase of particle concentration also increased the overall weight of the structure, resulting in less bending of the actuator in the upward direction due to gravity. Figure 10 shows expansions in length of the composites under different air pressure levels. The composite with low particle concentration expanded substantially due to the low structural stiffness as expected, whereas the composite with a high particle concentration changed its length little from the original length during actuation. 

## 4. Conclusions

We propose bilayer composites made of metal particles and elastomer and the manufacturing method based on a non-uniform magnetic field for soft bending actuators. The proposed method significantly simplifies fabrication of multi-layered composite structures, neither requiring complex design of the structure nor involving multiple steps of bonding of different materials. In this study, we fabricated bending actuators with different concentrations of nickel particles. During the curing process, a non-uniform magnetic field was applied to one side of the mold. The nickel particles were attracted toward the magnetic field, resulting in a bilayer composite. The actuation performance of the bilayer composite was characterized both in simulation and experimentally for bending and axial expansion. The result showed that the behavior was mainly dominated by the concentration of nickel particles. The proposed magnetically assisted fabrication for bilayer bending actuators will be a very useful technique for fast and robust manufacturing for various soft robotics applications.
